# A descriptive analysis of assessment measures on the effectiveness of a comprehensive stuttering intervention approach: A single case study

**DOI:** 10.4102/sajcd.v67i1.648

**Published:** 2020-04-29

**Authors:** Tasneem F. Karani, Anniah Mupawose

**Affiliations:** 1 Department of Speech Pathology and Audiology, Faculty of Humanities, University of the Witwatersrand, Johannesburg, South Africa

**Keywords:** stuttering intervention, person who stutters, case study, ICF, comprehensive approach, SSI-4, OASES, speech-language pathologist, South Africa

## Abstract

**Background:**

For effective client outcomes, stuttering assessment and intervention approaches need to be aligned. This encompasses using assessment and intervention approaches that address the three multidimensional constructs of stuttering, namely core behaviours, secondary behaviours and negative feelings and attitudes.

**Objective:**

The study aimed to explore whether multiple assessment measures could be used to describe the effectiveness of a comprehensive stuttering intervention approach, undergirded by the International Classification of Functioning, Disability and Health (ICF) framework.

**Method:**

A single-subject case design was employed with one male adult who stutters. Data was collected by administering the Stuttering Severity Instrument-Fourth Edition (SSI-4) and Overall Assessment of the Speaker’s Experience of Stuttering-Adults (OASES-A) at three testing periods (pre-intervention, immediately post-intervention and 7 months post-intervention), and a semi-structured interview schedule immediately post-intervention. Descriptive statistics was used to analyse the SSI-4 and OASES-A, and thematic analysis was conducted to evaluate the participant’s interview schedule responses.

**Results:**

The participant’s total scores, impact scores and severity ratings of both the SSI-4 and OASES decreased across the three testing periods. The main theme of effectiveness of the comprehensive stuttering intervention to reduce aspects of disability emerged from the participant’s responses.

**Conclusion:**

Evaluation of the results from the assessment measures revealed that the comprehensive stuttering intervention approach was effective in reducing the participant’s core behaviours, secondary behaviours and negative feelings and attitudes. Assessment and management of fluency disorders should promote a client-specific multidimensional approach that extends beyond the core behaviours and secondary behaviours, by addressing the underlying social and emotional facets of fluency disorders.

## Background

The purpose of this article was to demonstrate the effectiveness of a comprehensive stuttering intervention approach on an adult male participant who stutters based on multiple assessment measures. According to Bleek et al. (2012), stuttering is a multidimensional construct characterised by: (1) overt core behaviours, such as involuntary repetitions of syllables and words, prolongations of the sound(s) during the flow of speech and blocks of airflow, and (2) covert behaviours, a variety of behavioural, physiological, cognitive and emotional reactions to the speech disruptions. In essence, stuttering can be reduced to consisting of three components: (1) core speech behaviours, (2) secondary behaviours and (3) negative feelings and attitudes (Guitar, 2013). The interaction of these three components of stuttering often leads to debilitating effects. A person who stutters (PWS) may experience a variety of emotional challenges, which include sadness, shame, guilt, anger, resentment and fear (Yaruss, 2010; Yaruss & Quesal, 2006). This often leads to feelings of negative self-regard, such as self-blame and an overall sense of negative self-worth. These attitudes may cause further negative behavioural challenges such as avoidance of stuttering instances through secondary behaviours and avoidance of participation in activities that entail any communicative interactions (Yaruss, 2010; Bleek et al., 2012).

There are numerous studies that have explored the efficacy of using comprehensive intervention approaches that are intensive and based on the immersion principle (Blomgren, 2007; Kroll, 2010). The immersion principle is constructed on the premise that learning occurs when it is ‘intense’ and for shorter periods (i.e. ‘immersive’) (Blomgren, 2010). However, additional information is required to determine the success of non-intensive, traditional-based therapy that occurs over longer periods of time (i.e. weeks or months). Thus, this study set out to determine the efficacy of using a ‘whole-person’ stuttering approach on a South African multilingual male adult who stutters. The whole-person approach and comprehensive stuttering approach will be used interchangeably in this article. The effectiveness of this approach was determined using assessment measures that evaluate the three multidimensional constructs of stuttering.

The low incidence of fluency disorders recorded results in the collection of evidence-based outcomes being limited. In addition, individuals who stutter tend to also be heterogeneous because of their varied lived experiences. Thus, assessment measures and intervention approaches need to be adaptable to address the specific needs of a PWS. To that end, a single case study design was utilised in this study because it is known to be most appropriate to describe the effects of intervention. Moreover, single case designs consider the individual’s responses to a specific approach and are best suited to determine treatment efficacy as they closely resemble clinical practice (Millard, Nicholas, & Cook, 2008; Byun, Hitchcock, & Ferron, 2017).

As clinicians, it is vital to begin documenting the value of intervention approaches used to manage stuttering. Documenting stuttering interventions lends credence to what clinicians do and informs clinical practice. Additionally, the services provided need to subscribe to the principles of evidence-based practice as it is consistent with clinicians’ oath to client welfare (HPCSA, 2019). We argue that the effectiveness of stuttering interventions should be determined based on the assessment measure(s) that can evaluate the three multidimensional constructs of stuttering, namely core behaviours, secondary behaviours and negative feelings and attitudes. Indeed there has been a long-standing practice among clinicians to utilise assessment measures that assess stuttering frequency to determine the effectiveness of stuttering intervention (Yaruss, 2010). However, stuttering frequency is only a measure of one construct of stuttering (i.e. core behaviours). If comprehensive intervention approaches are implemented, then it follows that the assessment measures should evaluate the multidimensional nature of stuttering and also measure the generalisability of therapy techniques post-intervention (Blumgart, Tran, Yaruss, & Craig, 2012).

Arguably, South Africans have even a greater responsibility to provide services that redress the injustices of apartheid. An example of such an injustice was the inequitable access of speech-language pathology services to the majority of the black South African population who presented with communication disorders (Moonsamy, Mupawose, Seedat, Mophosho, & Pillay, 2017). Thus, as South African clinicians, it would behove us to continue to transform and use social justice as a value base for providing services that are attuned to the needs of the variety of populations that we service (Kathard & Pillay, 2013). Since South Africa consists of a rich diversity of people, languages and cultures, it is necessary for South African clinicians to be knowledgeable about and be willing to provide culturally and linguistically appropriate services (Mdlalo, Flack, & Joubert, 2016, 2019). With regards to using stuttering intervention that is comprehensive or involves the whole-person approach, it allows clinicians to be responsive to the heterogeneity (race, culture and language, among other factors) of the individuals who stutter that we serve.

The comprehensive stuttering approach addresses all three components of stuttering – core speech behaviours, secondary behaviours and negative feelings and attitudes. It utilises a combination of the fluency shaping (FS) and stuttering modification (SM) strategies and addresses the feelings and attitudes associated with stuttering. Fluency shaping is a behaviour modification approach that includes techniques such as easy onset, prolongations or stretching, and light articulatory contacts. Fluency shaping mainly targets changing the client’s overall speech pattern to obtain stutter-free speech, with little emphasis placed on the associated feelings and attitudes towards the stutter (Logan, 2015). Conversely, SM consists of four levels, namely identification, desensitisation, modification and stabilisation (Shapiro, 2011). Stuttering modification does not demand a PWS to obtain stutter-free speech. Rather, SM seeks to assist a PWS to learn to control or modify his or her stuttering and is coupled with addressing negative attitudes and feelings via cognitive restructuring or reframing to further improve fluency (Blomgren, 2010). Given the differences in the goals of the FS and SM approaches, along with the individual experiences of each PWS, the comprehensive or whole-person stuttering approach affords clinicians and the PWS greater flexibility to achieve success. In addition, such an approach is designed to increase the PWS’ feelings of confidence, subsequently improving his or her chances for effective participation in society (Shapiro, 2011).

The assessment measures and therapeutic outcomes of this intervention study were undergirded by the tenets of the International Classification of Functioning, Disability and Health (ICF), more commonly referred to as the ICF framework, as proposed by the World Health Organisation (WHO, 2001). The ICF provides a multidimensional framework through which a disorder such as stuttering can be described and evaluated to determine its impact on activity limitation and participation restriction (WHO, 2001; Yaruss & Quesal, 2006). According to the ICF, stuttering can be considered disabling due to its impairment on the body structures and functions (speech and respiratory musculature), its limitation on daily activities in relation to communication, and its restriction on participation in various contexts (Logan, 2015; WHO, 2001). The use of the ICF terminology provides clinician with a common language for clinical assessment and intervention of stuttering (Logan, 2015). The notion of assessing and evaluating intervention outcomes is being highlighted increasingly in the management of fluency disorders (Blomgren, 2010; Blumgart et al., 2012; Shapiro, 2011).

## Methods

### Objectives

1. To describe the scores obtained on the Stuttering Severity Instrument-Fourth Edition (SSI-4) with regards to its influence on core behaviours and secondary behaviours (impairments in body structures and functions), pre-intervention, immediately post-intervention and 7 months post-intervention.
2. To describe the scores obtained on the Overall Assessment of the Speaker’s Experience of Stuttering-Adults (OASES-A) with regards to its influence on activity limitations, participation restrictions and contextual factors (personal and environmental), pre-intervention, immediately post-intervention and 7 months post-intervention.
3. To describe the participant’s perspective on the influence of the comprehensive stuttering intervention on core behaviours, secondary behaviours and activity and participation in various contexts, immediately post-intervention.

### Research design

**FIGURE 1:**
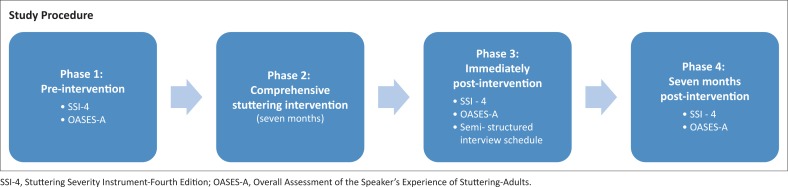
Four phases involved in data collection.

The study utilised a single-subject case design that included an intervention phase and an assessment phase, at three time periods (pre-intervention, immediately post-intervention and 7 months post-intervention) (Figure 1).

### Participant description

The PWS was a client at the University of Witwatersrand Speech and Hearing clinic. He was a 48-year-old, married, black male with two children. He worked as a steward for a well-established company. The PWS reported that the onset of his stutter was at 4 years old. He had previously received speech therapy during his primary school years, however, he reported receiving no benefit.

### Data collection

The instruments utilised at the three testing periods included the SSI-4 and the OASES-A. The SSI-4 provides a rating of the severity of the stuttering. This tool contains three sections: frequency (speaking and/or reading), duration and physical concomitants. The value of each section is matched to a task or scale score. To obtain the total score of the SSI-4 and the percentile ranking for the stuttering severity, the three sections need to be tallied up (Riley, 2009). The OASES-A assesses the impact of stuttering on an individual’s life. The OASES includes four sections – Section I: General information; Section II: Your reactions to stuttering; Section III: Communication in daily situations; and Section IV: Quality of life. For each section, an impact score is calculated and an impact rating is assigned. Thereafter, the impact scores of all four sections are tallied up to provide a total impact score (Yaruss & Quesal, 2006).

In phase 3, following the administration of the SSI-4 and OASES-A, a semi-structured interview schedule was conducted. It was used to obtain a broader description of the participant’s experience of stuttering and the comprehensive stuttering intervention approach, and to supplement information obtained from the SSI-4 and the OASES. Questions were generated based on the literature review and the OASES. A section of the interview schedule explored the effects of the stuttering approach on the participant’s core behaviours, secondary behaviours and feelings and attitudes (Appendix 1).

### Study procedure

#### Phase 2: Comprehensive stuttering intervention

Therapy was conducted at the university speech and hearing clinic by a student clinician. The student clinician completed a course in fluency disorders. Individual therapy sessions occurred once a week for a duration of an hour for 7 months (February–May and July–September). Therapy was terminated at the end of the academic year. The student was supervised for at least 15 min per session by a speech-language pathologist (SLP) registered with the Health Professions Council of South Africa. The expected outcomes of the therapeutic process were aligned with the domains of the ICF framework to minimise the debilitating effects of stuttering by addressing the impairments of body structures and functions, activity limitations and participation restrictions (WHO, 2001). Table 1 depicts the expected outcomes associated with the components of the ICF framework.

**TABLE 1:** Expected outcomes associated with components of the International Classification of Functioning, Disability and Health (ICF) framework.

ICF Component	Outcomes
1. Impairment (and associated loss of function)	Client will utilise a variety of fluency management techniques to improve his capacity to speak fluently with less motoric tension and involuntary movements: FS techniques of easy onset, prolongations or stretching, light articulatory contacts and pausing or phrasing. Successful Stuttering Management Programme (SSMP) (identification, desensitisation and modification). It should be noted that the SSMP phases were targeted in combination with FS techniques.
2. Activity limitations	Client will reduce the number and severities of activity limitations: SSMP (Identification and desensitisation) activities or situations that are avoided or feared. The SSMP was selected as this approach includes procedures that combine techniques targeted at desensitisation to stuttering, reducing avoidance behaviours, increasing acceptance of stuttering and motoric practices directed at reducing the tension associated with stuttering moments (Blomgren, Roy, Callister, & Merill, 2005). Utilise FS and SSMP speech techniques (cancellations, prep-sets, pull-outs) learnt in identified fearful activities.
3. Participation restrictions	Client will increase communication participation within and across daily activities and life roles (e.g. oral presentations at work, bible readings at church, etc.).
4. Personal factors	Client will reduce the impact of personal factors on verbal fluency: Develop strategies to manage negative feelings and attitudes. Develop constructive realistic attitudes towards stuttering, (i.e. acceptance).
5. Environmental factors	Reduce the impact of environmental factors on communication participation: Develop supportive listener reactions to client’s stuttering (e.g. client will educate significant others [employer spouse, colleagues, etc.] on stuttering).

*Source:* Adapted from Logan, K.J. (2015). *Fluency disorders*. San Diego, CA: Plural Publishing

ICF, International Classification of Functioning, Disability and Health; FS, fluency shaping; SSMP, Successful Stuttering Management Programme.

A typical session of the comprehensive stuttering approach included FS techniques, SM principles and activities to address the feelings and attitudes associated with stuttering, in order to limit activity limitations and participation restrictions (Guitar, 2013; Shapiro, 2011).

The sessions included the following:

- The therapist began the session by obtaining a 100-word conversational speech sample, which was recorded with the participant’s permission. The conversational sample was recorded at the beginning of each session in order to measure the percentage of syllables stuttered (SS) to allow for monitoring of therapeutic outcomes.
- The conversational sample was elicited by the client providing a recap of what was targeted in the previous session and how it was experienced by the client.
- Therapy would then proceed as per the ICF outcomes. Therapy session always targeted the three components of stuttering: core behaviours, secondary behaviours and negative feelings and attitude, depending on client’s progress and requests.
- Focusing on the client’s acceptance of stuttering.

#### Data analysis

Responses obtained from the SSI-4 and OASES were analysed using descriptive statistics, namely total scores, impact scores and severity ratings. Thematic analysis was utilised to analyse the participant’s responses from the semi-structured interview schedule.

### Ethical consideration

Ethical clearance certificate (STA-2016-40) was obtained from the non-medical research ethics committee of the University of the Witwatersrand, Johannesburg.

## Results

Results will be presented according to the three objectives of the study.

### Objective 1: Core behaviours and secondary behaviours (body structures and functions)

**FIGURE 2:**
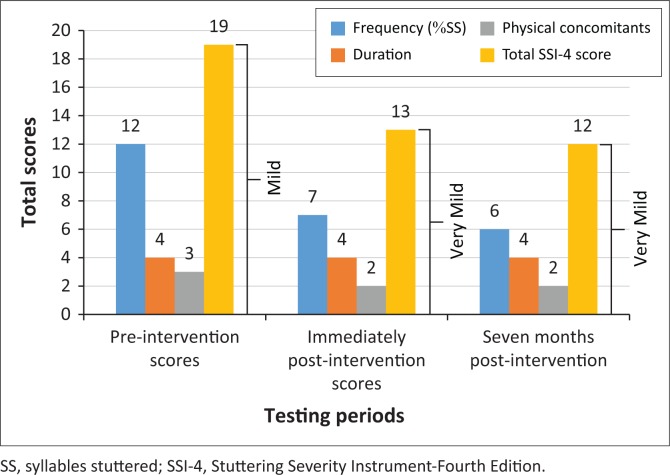
Total scores of the Stuttering Severity Instrument-Fourth Edition: Frequency, duration, physical concomitants and severity rating at three testing periods.

The severity of the participant’s stutter will be presented according to the total score of the frequency, duration and physical concomitants (secondary behaviours) at three testing periods (Figure 2).

Figure 2 reveals that the participant’s total scores of frequency decreased across the three testing periods. The severity rating also reduced from pre-intervention to post-intervention and then stabilised by third assessment interval. However, the duration (average severity of three longest stuttering events) (0.5 s–0.9 s) remained the same over the three testing periods.

### Objective 2: Activity limitations, participation restrictions and contextual factors

**FIGURE 3:**
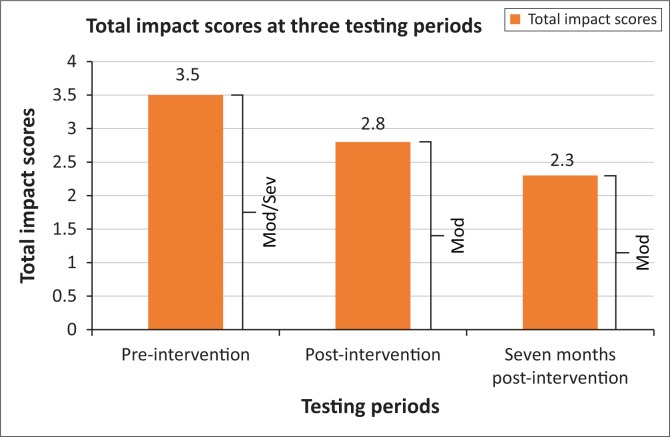
Participant’s Overall Assessment of the Speaker’s Experience of Stuttering results (total impact scores and severity ratings) at three testing periods.

The results of the OASES (total impact scores and severity) is presented in Figure 3.

The impact scores show a trend of scores decreasing, suggesting an improvement, from moderate/severe (mod/sev) to moderate (mod) across the three testing periods. For Section I (General information), the participant demonstrated improved knowledge regarding the speech processes which occur when he stutters (from *not at all*, to *very* when assessed immediately post-intervention, to *extremely* by the third testing). In Section II (Your reactions), the participant reported that he does not express himself as he pleases and uses filler words *often*, and this frequency was stated in all three testing periods. In addition, over the three testing periods, the feelings associated with stuttering (e.g. helpless, frustrated and anxious) reduced from feeling it *often* to *sometimes*. For Section III (Communications in daily situations), the participant expressed that pre-intervention, he found it *extremely difficult* to participate in social events. However, immediately post-intervention and 7 months post-intervention, he rated it as *not very difficult-somewhat difficult.* For Section IV (Quality of life), the participant changed his perspective of how much stuttering interfered with his educational opportunities from *a lot* to *a little*, to *not at all*, pre-intervention, immediately post-intervention and 7 months post-intervention respectively.

With regards to negative attitudes and feelings, the participant rated his self-acceptance on the therapist’s self-designed scale. He rated his self-acceptance from 1.5/5 to 4/5 over the course of the 7 months of intervention. His scores in the first half of therapy were variable depending on his communication experiences. However, scores started stabilising as therapy continued.

### Objective 3: Participant’s perspective on the influence of the comprehensive stuttering intervention approach on core behaviours, secondary behaviours and activity and participation in various contexts

The participant’s perspective on the influence of the intervention implemented is depicted in Table 2.

**TABLE 2:** Themes with their respective quotes from the participant on the influence of the comprehensive stuttering approach.

Theme	Sub-theme	Quote
Effectiveness of the comprehensive stuttering intervention approach to reduce aspects of disability	Core and secondary behaviours (body structures and functions)	‘… [*T*]herapy assisted in improving my speech.’ (Male, Steward, 48) ‘… [*S*]omething [*stuttering*] that I have done for years, it’s hard to change it overnight … I need more practice. So there are times when I forget …’ (Male, Stewart, 48)
	Activity limitations and participation restrictions	‘I feel it was on the whole person … the approach was more holistic … I think emotions … and once you are emotional nothing will happen. I think in terms of acceptance, it sort of helped to calm down.’ (Male, Steward, 48) ‘… It [participation] has improved a lot because I am more able to speak my mind in a meeting, at work and with my friends … I am now able to speak to a stranger on the phone. I am able to ask directions (laughs).’ (Male, Steward, 48)
	Personal factors	‘… [*A*]nd then I have gained much confidence and I am able to speak to anyone, … even those people who would have laughed and who I would’ve tried to avoid at all times …’ (Male, Steward, 48) ‘Knowledge of stuttering has help[ed] me to accept myself and have confidence that I can face the future without fear.’ (Male, Steward, 48) ‘I am not a stutter, I can’t be judged by my stutter. It’s not about how I say it, it’s about the content I’m conveying.’ (Male, Stewart, 48)

## Discussion

The discussion will elaborate on the three objectives of the study and their respective findings.

### Core behaviours and secondary behaviours (body structures and functions)

The results revealed that the total scores and severity ratings of the SSI-4 decreased with the comprehensive stuttering approach over the three testing periods. As the SSI-4 assesses the core and secondary behaviours, the scores suggest that using a combination of FS and SM techniques improved the client’s ability to speak fluently. Similar findings of improved speech fluency were reported by Euler, Lange, Schroeder and Neumann (2014). In their study, the 88 participants reported that FS, SM or a combination of FS and SM was more effective than other stuttering interventions that targeted core and/or secondary behaviours. This could be because FS and SM techniques are both effective at addressing the physiological and structural (impairment of the oral and respiratory musculature) aspects of stuttering that either predispose, precipitate or perpetuate the stutter (Shapiro, 2011).

However, it has been documented that there is a tendency for a PWS to default or relapse to old habits that facilitated the stuttering moments in the long-term, if no booster individual or group therapy is provided (Blomgren, 2010; Logan, 2015). This is due to the lack of contingencies placed on behaviour modification techniques (i.e. FS) used to increase speech fluency (Baxter et al., 2016).

### Activity limitations, participation restrictions and contextual factors

The results of the OASES showed a reduction in impact scores and severity ratings across the three testing periods. This demonstrated reduced activity limitations and increased communication participation. Similar findings were noted by Koedoot, Bouwmans, Franken and Stolk (2011), who compared the OASES scores of two groups of individuals who stutter. The groups included individuals who received stuttering therapy and those who did not receive stuttering therapy. There was a marked reduction in the impact scores and severity ratings of the individuals who received stuttering therapy. The OASES is seen as a good indicator of therapy outcomes more than that of stuttering severity related to frequency (Yaruss, 2010). Stuttering severity is more subject to variability as a PWS may use various strategies depending on the speaking situation and how the PWS has experienced the day or week (Constantino, Leslie, Quesal, & Yaruss, 2016). At times, there is a difference between how a PWS performs in the therapy room with a fluent-inducing stimuli (i.e. SLP), versus how they perform in other contexts such as work, social and home settings (Constantino et al., 2016).

With regard to attitudes and feelings, the results of the OASES indicated that the participant’s feelings and attitudes and overall participation improved. This was because of his improved overall psychological well-being, as depicted in Figure 3, by his OASES total impact scores and severity ratings across the three testing periods. These results are similar to those found by Klompas and Ross (2004) and Blumgart et al. (2012), which indicated from participants’ self-reports how stuttering intervention had improved their overall sense of well-being.

### Participant’s perspective

#### Effectiveness of the comprehensive stuttering intervention approach

The participant stated that the comprehensive stuttering approach positively influenced his life by addressing the ‘*whole person*’. Approaches that address the three facets of stuttering, core behaviours, secondary behaviours and feelings and attitudes, generally have good outcomes (Blomgren, 2010; Blomgren et al., 2005; Guitar, 2013; Logan, 2015).

#### Core behaviours and secondary behaviours (body structures and functions)

The participant stated that the comprehensive intervention approach assisted in *‘improving his speech*’. However, despite the improvement in the core behaviours and secondary behaviours noted in this study and in the literature, there is still an issue of relapse (Plexico, Manning, & DiLollo, 2005). Thus, it is important to evaluate therapeutic outcomes following the completion of therapy. The participant showed no relapse in therapy outcomes 7 months post-intervention. However, in the interview he brought to light the need for ongoing therapy to maintain the gains attained. Literature suggests that following the termination of therapy, booster individual and group therapy should be conducted to reduce the chances of relapse (Blomgren, 2010; Logan, 2015). A PWS may present with relapse following therapy termination (i.e. increase in speech dysfluencies), because ‘speaking normally’ may be too effortful of an outcome to achieve long-term (Logan, 2015). Hence, it is important to ascertain the goals of therapy with the PWS.

#### Activity limitations, participation restrictions and personal factors

The participant reported the positive influence of the comprehensive intervention approach on his feelings and attitudes and overall participation. He expressed: ‘Knowledge of stuttering has help[*ed*] me to accept myself and have confidence that I can face the future without fear’. The fact that the participant mentioned that he learnt to accept himself with the stutter is important to note as it indicates that he started acknowledging that stuttering does not define him as a person. It is not uncommon for a PWS to state that stuttering intervention was successful, even if there was minimal reduction in speech function (Blomgren, 2010, Logan, 2015). For some individuals who stutter, reducing activity limitations and participation restrictions can be achieved by improved self-acceptance. If a PWS has self-acceptance and self-confidence, they are willing to take on risks and engage in activities that they would never have otherwise.

## Conclusion

This study highlighted the importance of using a variety of assessment measures in evaluating and monitoring all three components of stuttering (i.e. core behaviours, secondary behaviours and feelings and attitudes) (Craig, Blumgart & Tran, 2009; Yaruss, 2010). The SSI-4 and OASES-A provided the quantitative measures, and the interview schedule showcased the individual qualitative gains. This allowed the client-specific nature of stuttering disorders to be highlighted and addressed. Furthermore, the comprehensive stuttering approach employed was effective in addressing all three components of stuttering. This was depicted by the reduced percentage of SS post-intervention, reduced impact scores on the OASES and the improved self-acceptance ratings.

Assessment and management of fluency disorders should practice a comprehensive or integrated approach that addresses the multidimensional nature of stuttering. Speech-language pathologists should draw on the theoretical framework of the ICF to provide therapy that extend beyond the core and secondary behaviours, and targets the underlying social and emotional facets of fluency disorders. The ICF framework provides clinicians and researchers with a more comprehensive perspective of the stuttering experience by considering aspects of ‘activity’ and ‘participation’ in therapy, while remediating the overt behaviours and improving covert behaviours (WHO, 2001). Current findings should be interpreted within the identified methodological limitations. Firstly, as this study employed a single case study design with a participant with a mild stutter, generalisability of the results to the wider population of individuals who stutter and those with greater severity ratings may be limited. However, despite the afore stated limitation, single case designs are more advantageous for use in the efficacy of interventions in clinical practice. Secondly, in critically transcribing the interview schedule, the identified theme and sub-themes may have been influenced by the researchers’ interpretations of the participant’s responses. However, the researcher utilised member checks. This was used to ensure clarity of the participant’s responses (Lodico, Spaulding, & Voegtle, 2010). Despite these stated limitations, the findings have significant implications on the practice of speech-language pathology, by affording clinicians with a more holistic view of a PWS, drawing on the ICF framework. In addition, this study provides clinicians with an overview of the structure and outcomes of the comprehensive intervention approach that can be utilised in the management of individuals who stutter.

It is recommended that further research expands on this study by increasing the sample size to include more individuals who stutter from various ethnic groups and ages. Further research should investigate the effectiveness of other approaches, being cognisant of intended areas of change and potential barriers to achieving long-term outcomes (Baxter et al., 2016). It is recommended that future studies include communication partners of individuals who stutter to obtain a broader perspective on the influence of stuttering and the efficacy of the comprehensive stuttering intervention approach on the core behaviours, secondary behaviours and feelings and attitudes, and overall participation of the PWS.

## Appendix 1

### Interview schedule (Participant [PWS])

#### Demographic information:

Age:

Gender:

Ethnic group:

Marital status:

Educational level:

Age of onset of stutter:

### 1. Activities and participation

#### 1.1. Education:

Describe your schooling experience. Has stuttering affected your schooling experience?

PROMPTS: Impact of stuttering, if applicable:

1. Schooling attendance, performance and overall academic success
2. Relationships and reactions: Teachers, peers and classmates
3. University or further studies: Career choices, performance

#### 1.2. Social life (friends and community):

Describe your social life. Has stuttering had any influence on your communication and social life?

PROMPTS: Impact of stuttering, if applicable

1. Reactions: Friends and community
2. Community and culture: View of stuttering and its causes
3. Interactions, participation and functioning in society (e.g. engaging in social activities-neighbours, church, making small talk at parties, telling jokes)

#### 1.3. Family:

Describe your relationship with your family members, spouse and children. Was it affected by your stutter?

PROMPTS:

1. Family history of stuttering
2. Family relationship: Parents, grandparents, siblings and extended family
3. Marriage: Decision to marry, courtship before marriage
4. Partner relationship: Partner power dynamics, children and your ability to discipline your children

#### 1.4. Employment:

Describe your employment experiences. Has stuttering had any influence on it?

PROMPTS:

1. Current employment: Job description, performance, satisfaction and promotions
2. Work relationships: Employer and co-workers

### 2. Feelings and attitudes

2.1. Describe your feelings and attitude growing up with a stutter
2.2. After receiving stuttering therapy, have these feelings and attitude about your stutter remained the same or changed, and in what way?

### 3. Speech therapy:

3.1. Tell me about your journey of speech therapy over the years and in particular the current speech therapy

PROMPTS:

1. Previous speech therapy: When, duration, approach used, view on the approach
2. Current speech therapy: Approach used and view on it. Has therapy affected you in any way? If so, how? Discuss its influence, if any, on stuttering behaviours, feelings and attitudes, and overall participation in everyday activities and life roles.
